# Digital dynamic radiography—a novel diagnostic technique for posterior shoulder instability: a case report

**DOI:** 10.1016/j.jseint.2023.02.015

**Published:** 2023-03-23

**Authors:** Zaamin B. Hussain, Sameer R. Khawaja, Anthony L. Karzon, Adil S. Ahmed, Michael B. Gottschalk, Eric R. Wagner

**Affiliations:** Department of Orthopaedic Surgery, Emory University, Atlanta, GA, USA

**Keywords:** Digital dynamic radiography, Dynamic imaging, Posterior shoulder instability, Posterior subluxation, Humeral head subluxation, Glenohumeral motion, Shoulder diagnosis

The range of motion (ROM) of the normal shoulder is a direct result of its few bony constraints and its reliance on static and dynamic soft tissue stabilizers. However, the trade-off is a predisposition to glenohumeral joint instability.^12^ The joint is statically stabilized by retroversion and articular congruity between the humeral head and glenoid, glenohumeral and coracohumeral ligaments, joint capsule, and labrum which deepens the glenoid fossa.^20^ Posterior shoulder instability encompasses patients with subtle subluxations to frank dislocations from a wide spectrum of pathologies.^9^^,^^13^ Structural abnormalities such as posterior glenoid bone loss or glenoid retroversion may predispose patients to posterior instability.^3^^,^^15^ Posterior shoulder instability accounts for 2%-10% of unstable shoulders and is commonly under- and misdiagnosed owing to its variety of clinical presentations.^2^^,^^5^^,^^17^ This low incidence compared to anterior shoulder instability is associated a paucity of studies compared to anterior instability and a resultant decreased understanding of posterior instability. Diagnosis is made radiographically in the setting of acute dislocations, whereas chronic instability is typically diagnosed with the presence of positive posterior instability provocative tests and can be confirmed with imaging studies showing posterior glenoid or labral pathology.^1^

Clinical diagnosis requires an experienced shoulder examiner to differentiate posterior instability from anterior, inferior, and multi-directional instability. Examination maneuvers such as the Jerk Test^1^ and the Kim Test^2^ have shown variable sensitivities and specificities for evaluating for this pathology. Complementing this, magnetic resonance imaging (MRI) is useful to see the static ligaments, labrum, and bony pathology.^10^^,^^11^ However, as an expensive and static imaging modality, it does not show the dynamic interplay of structures at glenohumeral joint during physiologic motion. Moreover, quantifying the severity of posterior instability is challenging and the lack of objective measurements makes it difficult to research and develop treatment algorithms.^16^ Furthermore, the challenges in evaluation of posterior instability likely underlies the challenges in treating this challenging pathology, with mixed results in open and arthroscopic treatments.^4^^,^^6^, ^7^, ^8^^,^^14^^,^^18^^,^^19^

With the implementation of digital dynamic radiography (DDR),^22^ we have improved our ability to better diagnose posterior shoulder instability and evaluate the extent of the pathology needed to correct in surgery. The purpose of this paper is to present this novel radiographic technique—and demonstrate its role in clinical workflow and how it overcomes the challenges associated with the diagnosis of posterior shoulder instability.

## Case report

A 28-year-old left-hand dominant male presented with left shoulder pain. He reported a history of over 200 left shoulder dislocations, over the preceding years. His surgical history consisted of multiple stabilizing reconstructive procedures including an anterior stabilizing procedure, capsulorrhaphy, and a posterior iliac crest bone grafting procedure, but the exact procedures were unknown and operative notes unavailable. All procedures had failed to correct his instability and led to continued subluxation/dislocation events. He reported dislocation events triggered by activities of daily living and having dislocation events in his sleep, with many reducing spontaneously but several requiring closed reduction in the emergency room. At the time of examination, he reported no numbness, weakness, or prior trauma.

Of note, he had a past medical history of generalized dystonia managed with deep brain stimulation device placement for the 10 years prior to our evaluation, complicated by battery failure, subsequent device non-functionality, and eventual implantation of bilateral neurostimulators. He was also previously managed with neuroleptic medications and upon neurology consult he was diagnosed with tardive dyskinesia as a medication adverse effect producing abnormal involuntary bodily contractions and tone, which was subsequently treated with botulinum toxin.

Upon examination, well healed surgical incisions were visualized over the anterior and posterior aspect of his shoulder. He had marked apprehension and his ROM examination was limited by pain. His left shoulder, marked by pain, had a flexion active ROM 50° and passive ROM 140°, abduction active ROM 50° and passive ROM 120°, external rotation active ROM 40° and passive ROM 70°, internal rotation (adduction) active ROM and passive ROM L5. Despite the discrepancy between active and passive ROM, he was neurovascularly intact indicating no evidence of a muscle or nerve injury on examination. The patient’s shoulder sat in a posterior laxed position, with a positive Jerk and Kim Test, and easily subluxated with any motion posteriorly or anteriorly.

**Figure 1:**
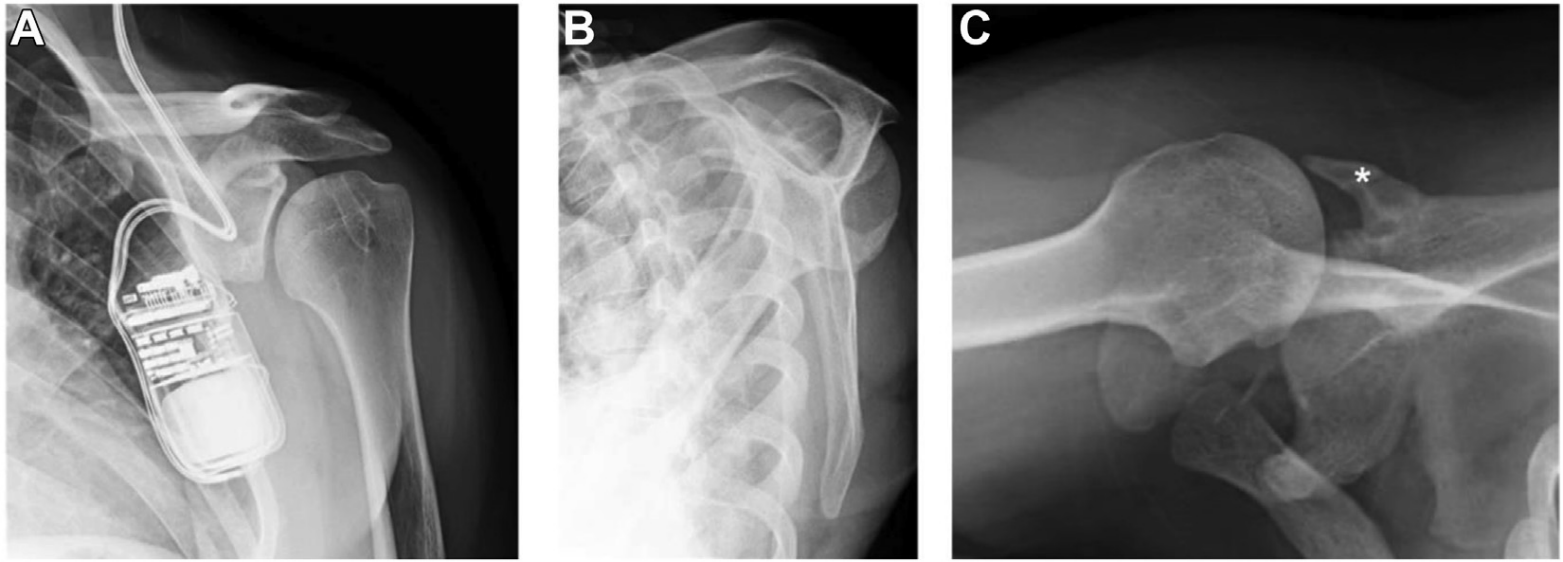
Plain film radiographs of the left shoulder (**A**) Grashey, (**B**) scapular Y, and (**C**) axillary. Significant posterior bone loss of the native glenoid with a posterior glenoid iliac crest bone graft (∗) can be seen. Lucency within the humeral head can be appreciated and resulted from prior procedures, and an implanted neurostimulator, which can be partially visualized.

**Figure 2:**
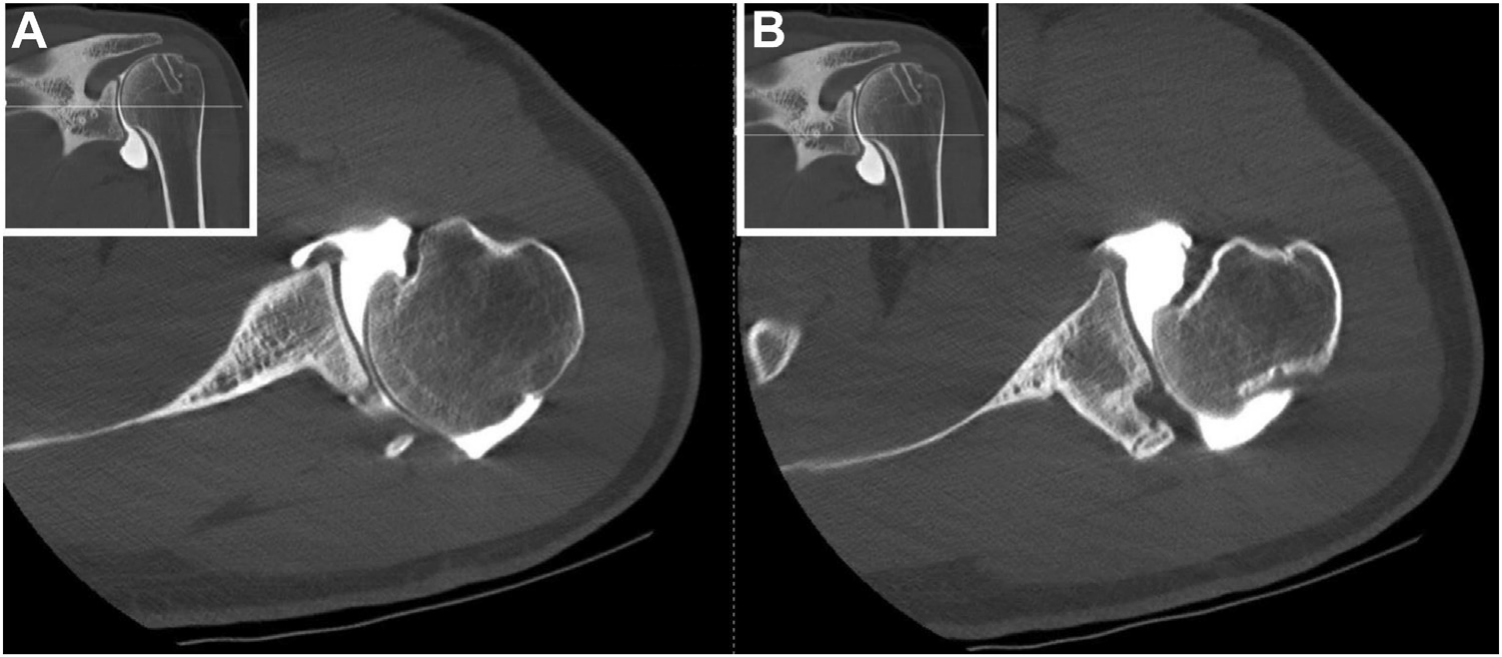
(**A**) CT arthrogram of the left shoulder demonstrating severe posterior subluxation of the humeral head, with absence of the superior, middle, and inferior glenohumeral ligaments. (**B**) Lucencies within the humeral head demonstrate removal of hardware from prior surgery. Presence of failed posterior iliac crest bone block graft, with chronic nonunion. *CT*, computed tomography.

Plain film radiographs of the left shoulder (Fig. 1) demonstrated substantial posterior bone loss of the native glenoid with a posterior glenoid bone graft. MRI was the preferred next diagnostic investigation; however, it was contraindicated due to implanted neurostimulators in this patient. As an alternative, a computed tomography (CT) arthrogram of the left shoulder was performed (Fig. 2), which demonstrated severe posterior subluxation of the humeral head and damage to the superior middle and inferior glenohumeral ligaments.

In the absence of MRI, ultrasound of the left shoulder was also performed to exclude any concomitant rotator cuff or scapular pathology which may have been contributing to the abnormal scapulothoracic dynamics. Subscapularis showed a mild attenuation with an upper 1/3 tear, but otherwise no other rotator cuff pathology was seen.

### DDR

Pulsed radiographs at 15 Hz for up to 20 seconds of left and right shoulder were taken. The entrance surface dose of radiation exposure for an average complete DDR shoulder exam is 1.33 mGy, which is comparable (∼1.3x) to the dose of a standard static 2-view shoulder x-ray exam. In order to standardize image acquisition, the dynamic images were taken under the supervision of one of three radiology technicians who coached patients on timing and cadence of the requisite shoulder motions. All patients were standing during image acquisition.

**Figure 3:**
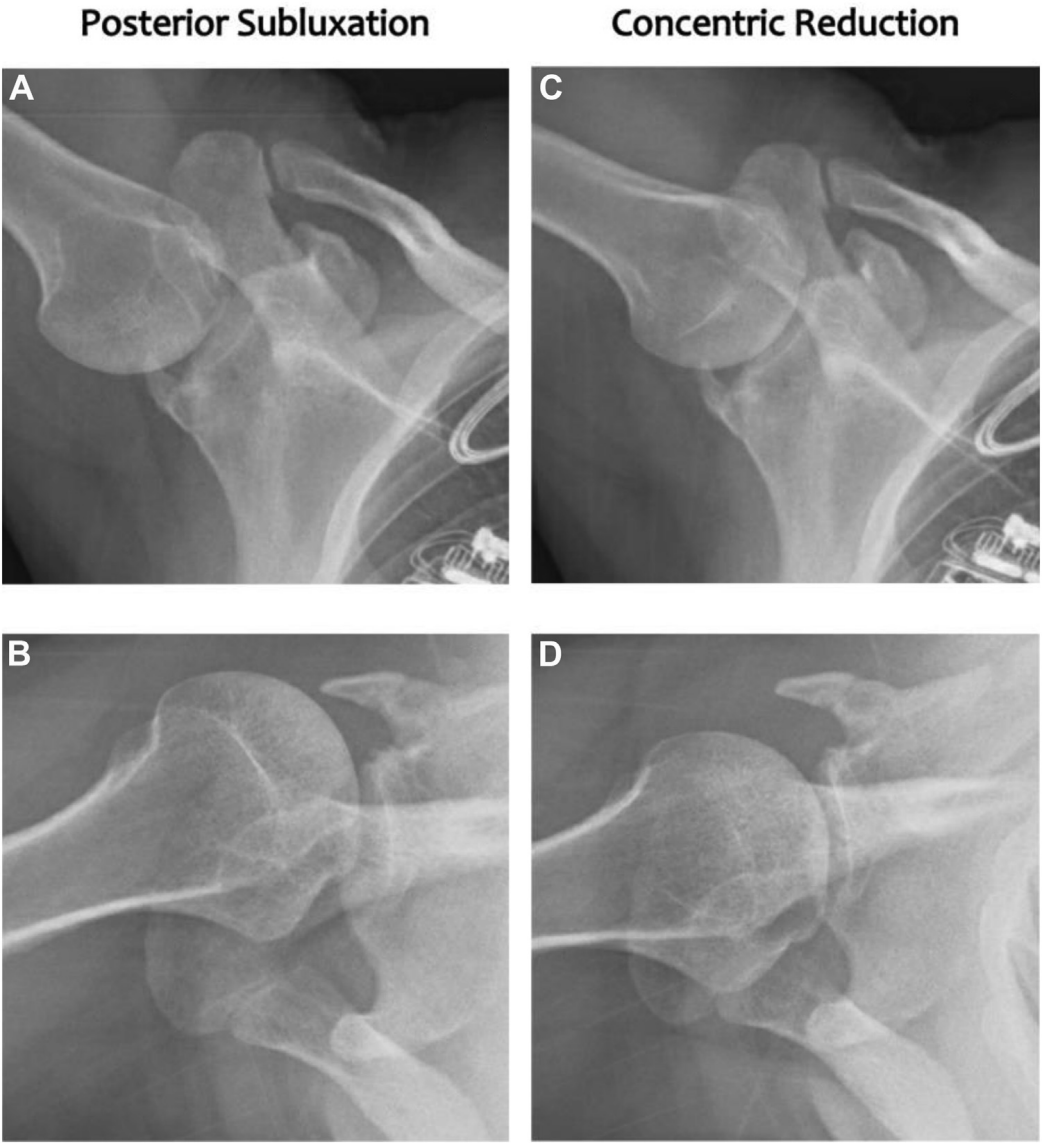
Static images of the digital dynamic radiography of the left glenohumeral joint. (**A**) Demonstrates a PA L shoulder XR and (**B**) an axillary view showing posterior subluxation. (**C** and **D**) With slight adduction, the humeral head concentrically relocates on corresponding views. *PA*, postero-antero; *L*, Lateral; *XR*, X-rays.

As demonstrated in Supplementary Video S1, DDR provides a direct visualization of the relationship of the humeral head and the glenoid under stress throughout the patient’s active ROM, thereby removing the diagnostic uncertainty of static imaging studies. Qualitatively, DDR revealed the humeral head severely subluxated posteriorly at rest (Fig. 3, Supplementary Video S1), and upon abduction the humeral head relocates congruently anteriorly.

## Discussion

This case report demonstrates a novel application of DDR for the diagnosis of posterior shoulder instability, which is notoriously challenging to diagnose with physical exam and static imaging modalities including MRI or CT arthrography as in this case.^17^ Currently, glenohumeral stability assessment and its associated treatments are guided by MRI or CT scans assessing ligament, capsular, and bony pathologic states. However, this is limited to a single point in time, without any ability to assess the glenohumeral joint dynamically through daily ROMs of the shoulder. It also does not allow for assessment of the contribution of scapulothoracic motions on a patient’s glenohumeral stability and pathologic process.

Integrating DDR into the clinical workflow allows dynamic non-invasive examination of shoulder kinematics, including glenohumeral and scapulothoracic motions.^22^ Indeed, Xiao et al have demonstrated the use of DDR and the role of scapulohumeral rhythm quantification in the diagnosis of shoulder pathology.^22^ Furthermore, this modality is an inexpensive method to objectively quantifying disease severity with a low radiation dosage. DDR provides a similar function to that of intraoperative fluoroscopy, particularly under the continuous “live” setting, except it is superior in several ways. Firstly, it allows image acquisition in the clinic setting and facilitates preoperative decision making before consuming precious operative time. For example, given this patient concentrically reduces the joint in certain motions, surgery was aimed at restabilizing the patient’s humeral head in order to stabilize the concentric reduction, instead of salvage procedures aimed solely at overcoming the glenoid bone loss. Secondly, a predesigned sequence of images allows for capturing the patients’ typical physiological ROM and it is reproducible, allowing for comparisons over time in the same patient and between patients. Finally, ionizing radiation is an important consideration, and the DDR sequences are designed to be extremely efficient and comparable (∼1.3x) to the dose of a standard static 2-view shoulder radiograph exam.

DDR has been described in other applications such as pulmonology and cardiology.^21^^,^^23^ However, this technology has the potential to make an important contribution to our understanding of dynamic pathologies in the musculoskeletal system which were traditionally elusive, such as scapulothoracic pathology, glenohumeral instability, and wrist kinematics.

There are some limitations in using DDR for the diagnosis of posterior shoulder instability. The diagnosis requires specialist equipment that is not currently widely available until recently. However, because of its versatility and its potential benefit in imaging in many joints throughout the body, this has the potential to be cost-effective and even replace more advanced imaging in certain pathologic states. At present, standardized protocols allowing radiographs to be taken at the same point in a joint’s ROM do not exist within an image series. This would allow direct comparisons between patients for research and is an area of current development. The quantification and the establishment of a severity grading scale based on the amount of subluxation is the natural progression of this technique. Furthermore, this imaging would give a true sense of the dynamic concept of “glenoid track” and which lesions are truly on and off track. Although the inability to perform an MRI in this case highlights the important role of DDR, it does somewhat limit our inferences. For example, MRI could have helped to establish the severity of the subscapularis tear seen on ultrasound as anterior rotator cuff insufficiency could also be contributing to the posterior instability. Additionally, it could also evaluate for a posteroinferior labral tear which cannot be seen on DDR, but could be a contributing pathology with an available solution.

## Conclusion

DDR can be used to assist with the diagnosis of shoulder instability, particularly in the posterior direction. Furthermore, it helps the surgeon to better understand the track of the humeral head, as well as if the head remains in a dislocated or concentrically reduced position. This case report is an example of how DDR can take a step forward in the evaluation of a difficult to treat pathology in the musculoskeletal system. It seems the future will likely involve other novel applications of this dynamic imaging assessment in other joints, enabling a more sophisticated assessment of the dynamic interplay of complex anatomical structures through a full range of joint motion.

## Disclaimers

Funding: The authors report no funding was used to complete the study of interest.

Conflicts of interest: Eric R. Wagner is a consultant for Stryker Corporation, Wright Medical Group N.V., Zimmer Biomet, Acumed, and Osteoremedies. He also receives research support from Arthrex and Konica Minolta. Michael B. Gottschalk receives research support from 10.13039/100008894Stryker Corporation, Konica Minolta, and 10.13039/100007307Arthrex. The other authors, their immediate families, and any research foundations with which they are affiliated have not received any financial payments or other benefits from any commercial entity related to the subject of this article.

Patient consent: Obtained.
